# Comparison of Piascledine (Avocado and Soybean Oil) and Hormone Replacement Therapy in Menopausal-Induced Hot Flashing

**Published:** 2011

**Authors:** Yunes Panahi, Fatemeh Beiraghdar, Nafise Kashani, Nika Baharie Javan, yahya dadjo

**Affiliations:** a *Chemical Injuries Research Center, Baqiyatallah University of Medical Sciences, Tehran, Iran.*; b *Nephrology and Urology Research Center, Baqiyatallah University of Medical Sciences, Tehran, Iran.*; c *Faculty of Medicine, Department of Genecology, Baqiyatallah University of Medical Sciences, Tehran, Iran.*; d *Faculty of Pharmacy, Islamic Azad University, Tehran, Iran.*

**Keywords:** Piascledine, Soy isoflavones, Phytoestrogens, Hormone replacement therapy, Climacteric symptoms, Hot flush

## Abstract

Different symptoms in Climacteric period, includes hot flash. Hormone replacement therapy (HRT) is common therapy for relief of menopausal symptoms but has possible contraindications and side effects. Recently Piascledine (combination of Avocado oil with Soybean oil) showed effects in reducing hot flash severity. Present study designed to compare the effects of HRT with Piascledine in treatment of hot flash.

The cases of this study were sixty-six women at the age range of 40 to 70 years and complaints of menopause-induced hot flashing, whose last menstruation dated at least 6 months prior to the beginning of the study. The patients in this open label clinical trial, randomized to receive Piascledine capsule 1 mg or HRT (0.625 mg oral daily Conjugated Estrogen tablets, plus 2.5 mg continuous oral daily Medroxyprogesterone Acetate tablets) for 2 month. Hot flash property and severity was assessed via a daily check list and Visual analog scale. Climacteric symptom was measured before and after intervention using Greene Climacteric Scale (GCS) and Blatt-kupperman Menopausal Index (BKMI).

Thirty-three eligible patients were allocated in each group. From the Piascledine group, one patient and from the HRT group, 16 patients weren›t willing to attend the study; therefore, 32 and 17 woman received treatment in Piascledine and HRT groups. 4 patients were withdrawn for vaginal bleeding and one for breast tenderness from HTR group. Hot flash severity in both groups decreased during the time similarly. With regard to GCS (p = 0.571) and BMKI (p = 0.891), the outcome was similar among the two groups.

Due to low HRT compliance and its possible risks in long period of time and considering the same activity of soybean supplement and HRT in relieving the hot flash as menopausal symptoms in women, it seems that soybean supplements can be an alternative therapy to hormone.

## Introduction

Majority of women complain different symptoms in Climacteric period, stage that women pass the fertility phase toward the menopause, includes hot flash that impact their quality of life. The hot flash is described as a heat or warmth sensation of the skin, often accompanied by other symptoms such as sweating (1).

Hormone replacement therapy (HRT) historically has been the most common therapies for relief of menopausal symptoms. According to possible contraindications (breast or endometrial cancer) and undesirable side effects (irregular bleeding, breast tenderness, thromboembolic events, mastalgia, nausea, migraine, weight gain and hydric retention), the compliance of the HRT is poor (2, 3).

Recently, using of herbal components especially the ones containing phytoestrogens for treatment of the climacteric symptoms have been noted (4). Phytoestrogens are naturally found in some vegetables which structurally and functionally similar to estradiol (3, 5, 6-8). There are four major classes of phytoestrogens: isoflavones, lignanes, flavonoids and cumestranes (9). The phytoestrogens with the most powerful estrogenic action are genistein, daidzein and glycitein which belong to the class of isoflavones and are found largely in soybeans and their by-products (3). Isoflavones are thought to exert both estrogenic and antiestrogenic effects, depending on the tissue (2, 10-12). Isoflavones may block the estrogen receptor (2, 10), thereby having an antiestrogenic effect on uterine and breast tissue (2, 13) where excess estrogen may promote tissue proliferation (2). Conversely, isoflavones may bind to the estrogen receptor (2, 5, 14, 15) and stimulate estrogen activity in other tissues, thereby having an estrogenic effect (2, 10).

Previous clinical trials showed different effects of soy isoflavones on vasomotor symptoms. An Australian study showed no significant difference in relief from menopausal symptoms in women consuming either soy flour or wheat flour (16). But a recent, double-blind, randomized, placebo controlled study of women with five or more hot flash episodes per day showed that the soy isoflavone was significantly superior to placebo, in reducing hot flash severity (15). Two trials of isoflavone pills have shown a modest benefit on vasomotor symptoms (8-19%) compared with controls (17, 18) whereas another did not detect any difference (19).

However the main question is whether soy isoflavones can be effective on climacteric symptoms especially hot flash and if so, can they provide a safe and effective alternative therapeutic for postmenopausal women.

The purpose of this study is to compare the effects of conventional HRT with Piascledine (Avocado oil unsaponifable 1 part, Soybean oil unsaponifable 2 parts) in treatment of hot flash as one of the important climacteric symptoms and also evaluate the safety of Piascledine in relieving postmenopausal symptoms.

## Experimental

This study was designed as open label, randomized, clinical trial and carried out in Baqiyatallah gynecologic outpatient clinics (Baqiyatallah Hospital, Baqiyatallah medical sciences university, Tehran, Iran) at May until October 2007. The Ethics Committee of Baqiyatallah Medical Sciences University approved the protocol and participants were handed in their informed consent before the study.

*Study participants*

Sixty-six subjects were included in the present study through inclusion criteria: postmenopausal women aged 40-70 years; last menstruation dated at least 6 months prior to the beginning of the study; follicle-stimulating hormone (FSH) Level greater than 20 IU/L, with complaints of hot flashes and written informed consent. Exclusion criteria were strict vegetarian, high-fiber or high-soy diet, intolerance to soy and history of breast cancer, endometrial carcinoma, cardiovascular disease, thromboembolic disorders, active liver or renal disease, chronic gastrointestinal diseases and undiagnosed vaginal bleeding. No subject was on hormone therapy or phytoestrogens within the preceding 6 months. Women were excluded from the study if they consumed drugs which caused and could interfere with the postmenopausal hot flashes (such as Niacin, Hydralazyin, Nitrates and Magnesium Sulfate). Women with contraindications or intolerance to conventional HRT were also excluded. Prior to the study, the thyroid stimulator hormone (TSH) and free tyrosine (T_4_) levels were measured to exclude thyroid dysfunctions that could interfere with the symptoms.

*Study design and randomization*

At baseline interview, data include: information on weight, height, arterial pressure, menarche, parity, time since menopause, menopause duration and also history of complete or partial hysterectomy, oophorectomy, poly cystic ovary syndrome (PCOS), hirsutism, acne and hair loss was collected.

A list was prepared before the beginning of the study. Women who had subsequent criterion for entering the study were randomly assigned to one of two groups A or B according to computer generated random number. Patients received the drug and were screened by the study recruitment personnel.

At baseline interview and at each follow-up session (every month) for each woman menopausal symptoms were evaluated using Hot Flash Questions (HFQ), Hot Flash Table (HFT), Visual Analog Scale (VAS) of hot flash severity, Blatt-kupperman Menopausal Index (BKMI) and Greene Climacteric Scale (GCS). Blood samples were collected from each subject after 12 h fasting at baseline and one and two months after treatment. All participants were instructed to record the daily number of hot flashes and severity of her hot flashes in a Daily Patient Questionnaire themselves and bring it to each visit (after one and two months of treatment). Participants were given a list of ‘foods to avoid’, which included legumes, soybeans and isoflavone supplements.

*Study intervention*

Group A, receiving 300mg oral daily Piascledine capsules (Avocado oil unsaponifable 1 part, Soybean oil unsaponifable 2 parts), Expanscience laboratories France. Group B, was received 0.625 mg oral daily Conjugated Estrogen tablets, plus 2.5 mg continuous oral daily Medroxyprogesterone Acetate tablets (Aburaihan pharmaceutical company, Iran).

*Outcomes measure*

HFQ contains four questions regarding the state of patient›s hot flash that asks them the following questions:

The average duration of their each hot flash experienced in a 24 h period, whether their hot flash wake them up from sleeping at night, if their hot flash prevents them from their usual activity during day time and whether they suffer from night sweating.

In order to estimate patient›s hot flash severity in this study, three different methods were used. The first method for hot flash severity investigation is the information that was acquired from Daily Patient Questionnaire. The second one is the number of activities that are done by patients due to hot flash. These activities were listed in HFT from number 1 to 5 questions as following:

Taking off their clothes; Cooling the place using cooling instruments; Taking cold shower; Placing ice on their skin for cooling; Drinking cold water.

Each of these questions was replied back as Yes/No by the patients. The patients that did more of these activities had encountered more severe hot flash.

The third way was VAS of hot flash severity that was designed as a 100-millimeter horizontal line without scaling (0 = no hot flash to 100 = unbearable hot flash problem), and the patients were asked to mark their current hot flash severity on it.

Blatt-kupperman Menopausal Index was based on Dr kupperman’s experience in treating women with menopausal complaints and is rooted in his desire to provide a numerical score for complaints which could then be used to evaluate treatments (20). This index is a numerical conversion system that grades 11 of the most common menopausal complaints, namely hot flushes, paraesthesia, insomnia, nervousness, melancholia, vertigo, weakness, arthralgia and myalgia, headache, palpitation and formication. Each symptom was rated on a scale from 0 to 3 for absent, mild, moderate and severe complaints. To calculate the BKMI, symptoms were weighed as follows: hot flashseverity (×4), paraesthesia, insomnia and nervousness (×2) and all other symptoms (×1) (20). Kupperman Menopausal Index is the sum of all 11 indices.

A new climacteric scale was constructed by Greene (21). This scale independently measures psychological, somatic and vasomotor symptoms and is presently used in studies in selected populations as a quality of life measurement (22). This scale measures a total of 21 symptoms. Each symptom is rated by the woman herself according to its severity using a four point scale: not at all (0); a little (1); quite a bit (2); extremely (3). Symptoms 1-11 address psychological symptoms divided in a measure of anxiety (a sum of symptoms 1-6) and of depression (a sum of symptoms 7-11). Somatic aspects are addressed in symptoms 12-18 and vasomotor symptoms are symptoms 19 and 20. Symptom 21 is a probe for sexual dysfunction. The total Greene Climacteric Score is the sum of all 21 scores (22, 23).

BKMI and also GCS were translated to Persian by back translation method. According to this method, original English version of BKMI and GCS were translated to Persian and then the Persian questionnaire were entrusted to another person who had not seen the main English documents at all and the Persian samples were translated back to English once more and compared with the presented original one. The original and the back translated documents were the same.

Adherence, tolerability and adverse effects of each treatment were assessed by means of a symptom questionnaire and adverse events reports (ADR) during clinical visits.

*Statistical analysis*

Sum of actions that listed in HFT, VAS of hot flash, BKMI, GCS and subscales at the beginning of the study were subtracted from middle and end of treatment phase to calculate changes due to the intervention. These changes were compared between study groups with t-test (after passing normality assumption) and Mann-Whitney (when normality assumption was rejected). The chi-square test was used to compare problems due to hot flash. Data were presented as mean ± standard deviation and frequency (percentage) and analysis was done in SPSS 13 (SPSS Inc. Chicago IL.).

## Results and Discussion

This study was performed on 66 eligible patients who were divided into two groups. Each group contained 33 patients. From the Piascledine group, one patient and from the HRT group, 16 patients weren›t willing to attend the study that among these 16 patients, 10 of them did not attend because of fear from appearing cancer and also, 6 of them did not attend because of fear from other adverse effects due to HRT such as vaginal bleeding.

During the study, none of the patients from Piascledine group left the study but in second group, 3 patients because of vaginal bleeding and 1 patient because of breast tenderness were excluded from the study and 2 patients were discontinued treatment due to fear from appearing cancer and also, 2 patients didn›t answer the callings during the first month. During the second month in the second group, 1 patient was excluded because of vaginal bleeding, 1 patient discontinued the treatment because of fear from appearing cancer, and 1 patient did not answer the callings. Total number of patients at the end of study for Piascledine group was 32 and for HRT group was 6.

As shown in Table 1, baseline characterizes of patients of two groups didn’t have any significant difference.

**Table 1:** Baseline data

		**Piascledine (n = 32)**	**HRT (n = 17)**	**p-value**
		**Mean ± SD**	**Mean ± SD**	
**Age (year)**		53.0 ± 5.5	51.2 ± 6.0	0.307
**BMI (k/m2)**		27.9 ± 4.4	26.3 ± 3.7	0.249
		n (%)	n (%)	
**Education Level**	Lower Diploma	13 (40.6%)	8 (47.1%)	0.665
	Diploma and up	19 (59.4%)	9 (52.9%)	
**Occupation**	Housekeeper	23 (71.9%)	12 (70.6%)	0.924
	Employee	9 (28.1%)	5 (29.4%)	
**Pregnancy count**	1 to 3	26 (81.3%)	10 (58.8%)	0.826
	4 and more	16 (18.8%)	7 (41.2%)	
**Menopause Duration**	lower 2y	10 (31.3%)	10 (58.8%)	0.062
	more than 2y	22 (68.8%)	7 (41.2%)	
**Comorbidity**	No	14 (43.8%)	5 (29.4%)	0.327
have		18 (53.6%)	12 (70.6%)	
**Menopause Drug**	Yes	16 (50.0%)	8 (47.1%)	0.845
	No	16 (50.0%)	9 (52.9%)	
**Gynecological symptoms**	Hysterectomy	8 (25.0%)	2 (11.8%)	0.274
	Hirsutism	2 (6.3%)	4 (23.5%)	0.164
	Voice	0 (.0%)	0 (.0%)	-
	Alopecia	6 (18.8%)	4 (23.5%)	0.693
	Acne	2 (6.3%)	1 (5.9%)	0.953
	PCO	3 (9.4%)	5 (29.4%)	0.071

Based on comparing the information acquired from HFQ at the beginning and at the end of study for investigating hot flash state in both groups, most of the problems due to hot flash were reduced by using the drug and this reduction was almost the same in both groups. Before the beginning of the study, all the patients in both group had hot flash with the period of more than 6 min. At the end of the study, 2 patients (33.3%) in HRT group and 9 patients (28.1%) in Piascledine group had this problem in such manner (p = 0.796).

Before the beginning of the study, 12 patients (37.5%) in Piascledine group and 15 patients (88.2%) in hormone group woke from sleeping due to hot flash (p = 0.001) that at the end of the study, 3 (9.4%) and 2 (33.3%) patients were still suffered from this problem in Piascledine and hormone groups respectively (p = 0.111). Prevention from usual activity during day time (p = 0.949) and night sweating (p = 0.671) are also equal after treatment in both groups (Table 2).

**Table 2:** Comparison of problems due to hot flash in studied groups during the study base on HFQ.

**Hot Flash problems**	**Group**	**Baseline**	**p-value**	**After 1 month**	**p-value**	**After 2 month**	**p-value**
**HF Duration ** **(6 min and more)**	Piascledine	32(100.0%)	---	18(56.3%)	0.242	9(28.1%)	0.796
	HRT	17(100.0%)		7(77.8%)		2(33.3%)	
**Wake up from sleeping due to hot flash**	Piascledine	12(37.5%)	0.001	5(15.6%)	0.014	3(9.4%)	0.111
	HRT	15(88.2%)		5(55.6%)		2(33.3%)	
**Prevention from usual activity due to hot flash **	Piascledine	24(75.0%)	0.100	9(28.1%)	0.353	5(15.6%)	0.949
	HRT	16(94.1%)		4(44.4%)		1(16.7%)	
**Night sweating**	Piascledine	26(81.3%)	0.057	12(37.5%)	0.706	8(25.0%)	0.671
	HRT	17(100.0%)		4(44.4%)		2(33.3%)	

The number of patients that showed the symptoms and percentage of them were mentioned. At the end of study 6 and 32 patients remained at HRT and Piascledine groups respectively

**Figure 1:**
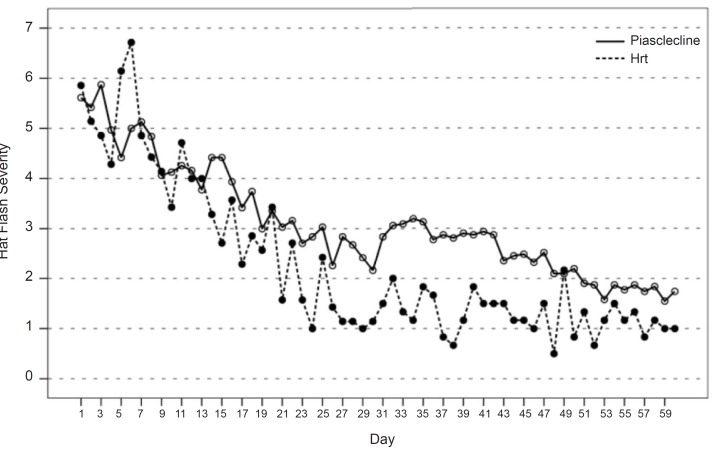
Hat flash severity during the study base on Daily pation questiunnarie

The process of daily hot flash severity index during the study based on the acquired information from Daily Patient Questionnaire has been shown in Figure 1. As shown, based on patients’ declaration, hot flash severity in both groups had been decreased during the time. Before the beginning of the study, this severity had an amount of 6 units in both groups and at the end of study, was 1 in hormone group and nearly 2 in Piascledine group. But this reduction had similar process in both groups.

Based on the acquired information from HFT, the number of activities which were done as a result of hot flash was 1.83 ± 1.47 and 1.31 ± 0.78 unit reduction in hormone and Piascledine groups, which was not statistically significant (p = 0.493). Also, the VAS of hot flash severity decreased 44.17 ± 20.60 and 39.78 ± 23.57 in hormone and Piascledine groups without significant difference (p = 0.800).

Blatt-Kupperman index which is a scale of measuring the postmenopausal symptoms in women, had a reduction of 8.50 ± 4.83 in Piascledine and 8.33 ± 4.89 in hormone groups which was statistically similar between both groups (p = 0.984). The total grade of GCS index (p = 0.571), psychological problems (p = 0.399), anxiety (p = 0.185), depression (p = 0.770), somatic aspects (p = 0.830), vasomotor symptoms (p = 0.682) and sexual activity (p = 0.422) had not significant difference between studied groups (Table 3).

**Table 3:** Comparison of change in Hot flash severity base on HFT and VAS and menopausal symptoms during the study base on BKMI and GCS

	**Time duration**	**Piascledine**	**HRT**	**p-value**
**Hot Flash severity**				
Activates done by patients to relief their hot flash	Mid - Before	1.06 ±.84 (1.0)	1.56±1.33 (1.0)	0.410
	End - Before	1.31 ±.78 (1.0)	1.83±1.47 (1.5)	0.493
VAS	Mid - Before	25.41 ± 19.87 (22.5)	34.44±20.53 (25.0)	0.255
	End - Before	39.78 ± 23.57 (42.5)	44.17±20.60 (42.5)	0.800
**BKMI**				
Un-weighted	Mid - Before	4.88 ± 3.83 (4.0)	5.56±4.48 (4.0)	0.816
	End - Before	8.50 ± 4.83 (8.0)	8.33±4.89 (8.0)	0.984
Weighted	Mid - Before	8.91 ± 6.44 (8.0)	10.44±7.49 (8.0)	0.631
	End - Before	15.28 ± 7.87 (14.0)	15.17±9.87 (12.5)	0.891
**GCS**				
Psychological	Mid - Before	4.50 ± 4.61 (4.0)	5.67±5.55 (4.0)	0.745
	End - Before	7.00 ± 5.72 (6.0)	9.17±6.43 (9.5)	0.399
Anxiety	Mid - Before	2.31 ± 2.25 (2.0)	3.11±3.72 (1.0)	0.963
	End - Before	3.56 ± 2.76 (3.0)	5.67±3.93 (5.5)	0.185
Depression	Mid - Before	2.19 ± 2.86 (2.0)	2.56±2.70 (2.0)	0.609
	End - Before	3.44 ± 3.65 (2.0)	3.50±3.15 (2.5)	0.770
Somatic aspects	Mid - Before	2.78 ± 2.64 (2.0)	2.89±2.52 (2.0)	0.841
	End - Before	4.28 ± 3.31 (4.0)	4.67±3.33 (5.0)	0.830
Vasomotor symptoms	Mid - Before	1.75 ± 1.70 (1.5)	2.11±1.69 (2.0)	0.546
	End - Before	2.84 ± 1.78 (2.0)	3.17±1.60 (2.5)	0.682
Sexual Interest	Mid - Before	0.13 ± 0.71 (.0)	0.33±0.71 (.0)	0.525
	End - Before	0.41 ± 0.84 (.0)	0.67±0.82 (.5)	0.422
Total Scale	Mid - Before	9.16 ± 7.76 (8.0)	11.00±8.70 (8.0)	0.588
	End - Before	14.53 ± 8.84 (13.5)	17.67±10.93 (17.0)	0.571

Data presented as mean ± Standard deviation (median). Before: Before treatment, Mid: One month after treatment, End: Two month after treatment

Biochemical parameters during the time of study has been shown in Table 4, which had not significant change whether before the beginning or at the end of the study.

The results of present study demonstrated comparable effects of Piascledine in decreasing the severity of hot flash and related problems as revealed in the HFQ, HFT and VAS, while there were no significant difference between Piascledine and HRT treated groups in decreasing the signs of hot flash and related problems.

**Table 4:** Comparison of biochemical factor in study group during the study

	**Time duration**	**Piascledine**	**HRT**	**p-value**
FBS (mg/dL)	Before	95.2 ± 9.5	98.7 ± 17.6	0.908
	Mid	93.1 ± 12.3	96.6 ± 9.4	0.618
	End	95.4 ± 8.9	94.0 ± 10.4	0.981
Cholesterol (mg/dL)	Before	219.7 ± 31.7	196.9 ± 49.1	0.376
	Mid	218.6 ± 36.4	214.3 ± 47.7	0.984
	End	220.7 ± 28.7	200.4 ± 51.8	0.448
HDL (mg/dL)	Before	56.2 ± 11.9	50.4 ± 14.5	0.219
	Mid	59.0 ± 19.6	57.6 ± 18.9	0.921
	End	61.9 ± 15.8	60.8 ± 17.3	0.752
LDL (mg/dL)	Before	129.2 ± 29.2	98.8 ± 44.3	0.110
	Mid	137.2 ± 34.0	115.0 ± 41.6	0.253
	End	131.8 ± 29.7	109.0 ± 43.1	0.391
TG (mg/dL)	Before	153.3 ± 71.0	176.2 ± 162.8	0.512
	Mid	140.8 ± 47.6	196.8 ± 166.7	0.636
	End	165.7 ± 71.9	177.2 ± 195.9	0.129
T4 (ng/mL)	Before	95.6 ± 18.5	96.6 ± 17.4	0.983
	Mid	88.1 ± 25.8	75.6 ± 30.7	0.313
	End	88.8 ± 16.1	87.3 ± 9.6	0.978
T3 (ng/mL)	Before	1.1 ± 2.5	1.4 ± .2	0.685
	Mid	1.2 ± .3	1.3 ± .3	0.100
	End	1.5 ± 1.9	1.5 ± .4	0.262
TSH (μIU/mL)	Before	2.7 ± 2.7	2.5 ± 1.0	0.238
	Mid	2.3 ± 2.5	1.8 ± 1.1	0.915
	End	1.9 ± 1.7	2.1 ± 1.6	0.602
FSH (IU/L)	Before	76.2 ± 37.4	75.3 ± 38.3	0.985
	Mid	81.0 ± 33.8	86.6 ± 48.4	0.995
	End	74.4 ± 30.8	70.4 ± 46.6	0.732
LH (IU/L)	Before	29.7 ± 14.8	32.8 ± 11.2	0.446
	Mid	33.3 ± 11.6	30.2 ± 15.1	0.537
	End	33.0 ± 11.6	29.2 ± 6.3	0.631
17B-est (pg/mL)	Before	57.9 ± 51.8	67.7 ± 30.3	0.189
	Mid	43.3 ± 32.3	91.1 ± 73.4	0.055
	End	56.7 ± 51.5	113.0 ± 92.7	0.731

Data presented as mean ± Standard deviation. Before: Before treatment, End: Two month after treatment. At the end of study 6 and 32 patients remained at HRT and Piascledine groups respectively

Hormone replacement therapy is applied to relieve vasomotor symptoms (24). Despite the apparent benefits of HRT with regard to the management of menopausal symptoms and prevention of degenerative disorders associated with the menopause (25) The uptake of HRT is less than 20% of women over 65 were not receiving hormonal components for preventing, and treating osteoporosis and cardiovascular disease (26, 27) and Many women are reluctant to initiate HRT because of concerns regarding the risks and side effects such as irregular bleeding, breast tenderness, mastalgia and risk of breast cancer after long term exposure to HRT (26-29). In addition, 25% of patients stopped therapy within 6 months (25) and approximately 70% of the women discontinued HRT after the first year of treatment (32). Low desire for receiving HRT from patients and deciding not to participate in the study was mostly due to concern about breast cancer. Also, low compliance of patients for HRT and stopping the treatment was mostly due to vaginal bleeding in this study, which is the same reason as other reported results in the similar studies. In addition to low compliance of HRT among women, some of them has absolute contraindications (breast cancer, endometrial cancer, undiagnosed vaginal bleeding, and thrombo-embolic diseases) or partial contraindications (history of thrombo-embolic diseases, systemic lupus erithrematose, athrosclerosis, and malignant melanoma) toward using HRT (25).Woman›s Health Initiative (WHI) reported that using HRT caused to 26% increase in the risk of breast cancer, 29% increase in the risk of coronary heart disease and 41% increase in the risk of stroke(25).

*Study by Ettinger et al. *has showed that among treatment side effects, vaginal bleeding was the most frequently reported reason for stopping HRT; it was the primary reason for stopping in 52% of older women and 29% of younger women (28). In another study which was carried out in Turkey 28.7% of the women received HRT for mean of 4.5 months and then stopped it mostly due to vaginal bleeding (33). Another study in Iran showed that the protocol of HRT treatment was not complete in 34.8% of cases and the most common reason for being stopped HRT was vaginal bleeding (34). The vaginal bleeding also, in HRT group of the present study, was the most common reason for stopping the treatment and on other hand 6 of 33 in HRT patients did not attend because of fear from adverse effects due to HRT such as vaginal bleeding. Treatment with Piascledine had a remarkable tolerability and it was revealed by the patients of this group that continued to treatment and any of them were not left the study.

In the study conducted by *Ettinger et al.*, 7% of younger women and 1% of older women discontinued HRT due to concern about breast cancer (29) and also in a study conducted in Iran, cancer was considered as the reason which resulted to stop using HRT (34). In accordance to the mentioned studies, in present study, concerning from cancer was evaluated as second reason for stop continuing of treatment. In addition similar to results of present study that showed the breast tenderness as a reason to discontinue the hormone replacement therapy, there was other study that finds breast tenderness in women to stop treatment (29).

In attention to low tendency of patients for beginning the consumption of HRT, low compliance of women in continuing HRT, several reasons for absolute and relative contraindications and also probabilities side effects of long consumption period, alternative HRT therapies are attended by physician and patients (24, 31, 35). One of these alternative HRT treatments is Soy phytoestrogen supplements (Isoflavons) (36). These natural phytoestrogens has estrogenic characteristics and are able to bind to estrogen receptors that lead to their agonist-antagonist characteristic depends on different target tissues (9, 37, 38). In the recent years, Soybean phytoestrogens have got attention of many researchers due to their potential effects in preventing cardiovascular diseases, post menopausal signs, cancer, and osteoporosis (24). Soy food and Soy isoflavons are being used by some women as natural alternative hormone therapies for relieving the menopausal symptoms (36).

Most of the studies were comparing soybean isoflavons with placebo, but the present study compared isoflavons effects on the vasomotor symptoms with HRT. In the present study, severity and duration of each hot flash had a same decreasing in both Piascledine and hormone groups. Also, the problems due to hot flash in both groups decreased as the same. Improvement of psychological, somatic, vasomotor problems, and sexual activity and Blatt-kupperman results in both groups were the same. Another study that compared isoflavone and HRT concluded that isoflavones’ effect on climacteric symptoms was similar to that from estrogen. Also soy isoflavone has no effect on endometrium and vaginal mucosa during the treatment (39).

In the present study, there was no side effect during treatment in Piascledine group and in the treatment period, it was well tolerated by patients while in the hormone group incidence of side effects such as breast tenderness and vaginal bleeding resulted to discontinue the drug consumption and may due to poor compliance of hormone therapy. The results of a study showed high compliance to soybean isoflavons in patients, similar to the study that reported tablets containing these extract were well tolerated with no serious problem related to isoflavons therapy (15).

One important limitation of present study is related to the higher rate of patient’s cooperation with the study at HRT treated group as seen as the decreased number of patients at the end of study that decrease the value of data. Future studies with large number of cases may help overcome this problem and obtain exact profile of the effects of HRT in comparison to the Piascledine. The results of Piascledine group by its own confirmed the beneficial effects of this treatment and suggested it as a useful supplementary treatment for hot flash in clinic.

Briefly, desire for consuming phytoestrogens among women is more than synthetic estrogens so low tendency to synthetic estrogens can be probably due to high collapse in hormone group in the present study. Due to low HRT compliance and its possible risks in long period of time in one hand and the similar activity of soybean supplement and HRT in relieving the menopausal symptoms on the other hand, it seems that soybean supplements can be an alternative therapy to hormone. However, the mentioned collapse in the present study and low participants made judgment difficult; therefore further studies with large number of patients are required to precisely determine the Piascledine as a reasonable alternative.
